# Supplementary material for: Association between oral microbiome and seven types of cancers in East Asian population: a two-sample Mendelian randomization analysis
Source: Front Mol Biosci. 2023 Nov 21;10:1327893. doi: 10.3389/fmolb.2023.1327893 (PMC10702768; doi:10.3389/fmolb.2023.1327893)
Supplement: Supplementary file 1 [file DataSheet1.ZIP › Supplementary Materials/MR plots for tongue/tongue═╝/Colorectal cancer/pheno.465_to_colorectal cancer_forest.pdf]

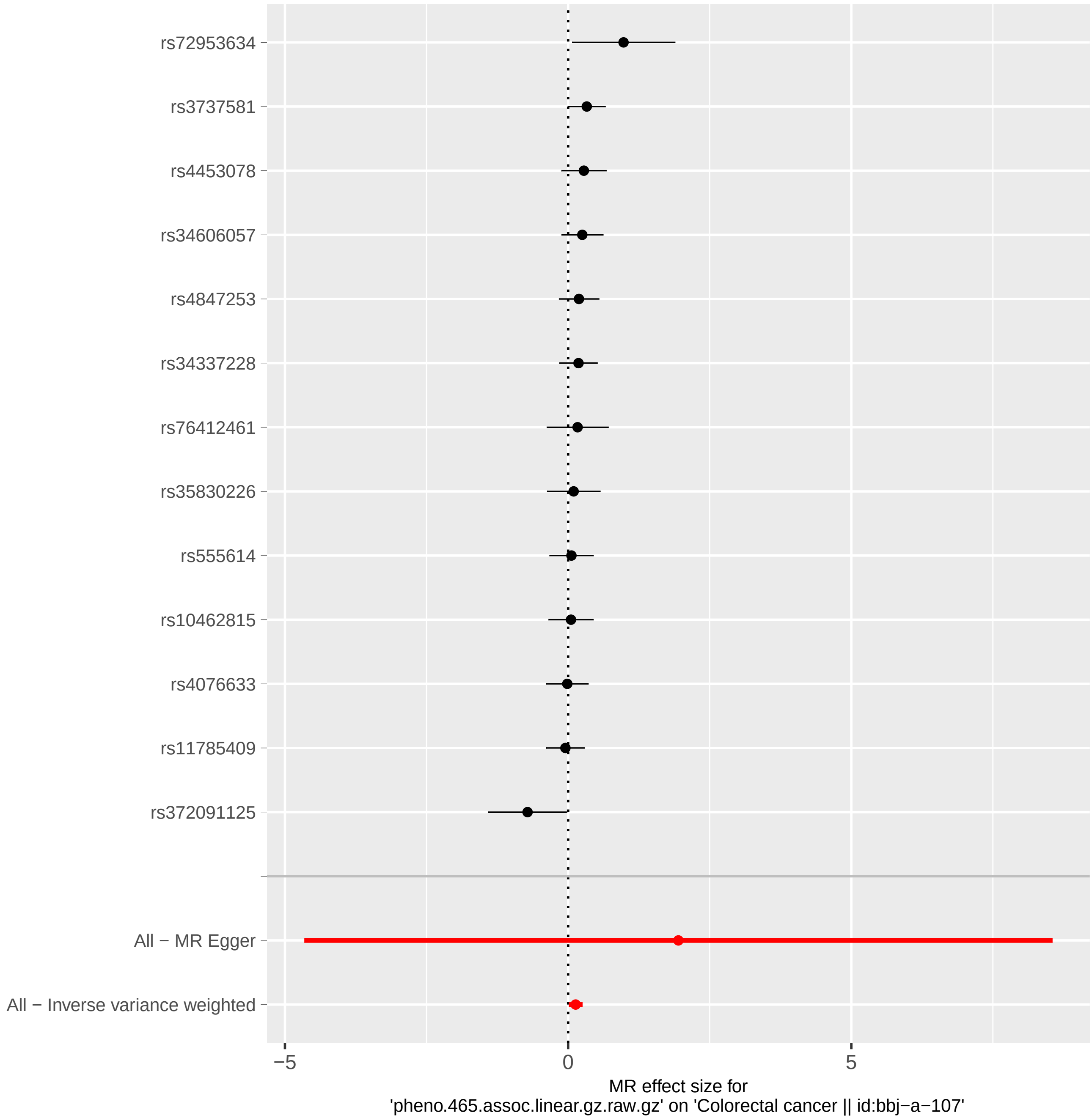
rs72953634
rs3737581
rs4453078
rs34606057
rs4847253
rs34337228
rs76412461
rs35830226
rs555614
rs10462815
rs4076633
rs11785409
rs372091125
All − MR Egger
All − Inverse variance weighted
−5
0
5
MR effect size for
'pheno.465.assoc.linear.gz.raw.gz' on 'Colorectal cancer || id:bbj−a−107'
